# Sex Differences in the Development of the Rodent Corticolimbic System

**DOI:** 10.3389/fnins.2020.583477

**Published:** 2020-09-30

**Authors:** Hanista Premachandran, Mudi Zhao, Maithe Arruda-Carvalho

**Affiliations:** ^1^Department of Psychology, University of Toronto Scarborough, Toronto, ON, Canada; ^2^Department of Cell and Systems Biology, University of Toronto Scarborough, Toronto, ON, Canada

**Keywords:** development, hippocampus, amygdala, prefrontal cortex, sex differences, corticolimbic, gonadal hormones, puberty

## Abstract

In recent years, a growing body of research has shown sex differences in the prevalence and symptomatology of psychopathologies, such as depression, anxiety, and fear-related disorders, all of which show high incidence rates in early life. This has highlighted the importance of including female subjects in animal studies, as well as delineating sex differences in neural processing across development. Of particular interest is the corticolimbic system, comprising the hippocampus, amygdala, and medial prefrontal cortex. In rodents, these corticolimbic regions undergo dynamic changes in early life, and disruption to their normative development is believed to underlie the age and sex-dependent effects of stress on affective processing. In this review, we consolidate research on sex differences in the hippocampus, amygdala, and medial prefrontal cortex across early development. First, we briefly introduce current principles on sexual differentiation of the rodent brain. We then showcase corticolimbic regional sex differences in volume, morphology, synaptic organization, cell proliferation, microglia, and GABAergic signaling, and explain how these differences are influenced by perinatal and pubertal gonadal hormones. In compiling this research, we outline evidence of *what* and *when* sex differences emerge in the developing corticolimbic system, and illustrate how temporal dynamics of its maturational trajectory may differ in male and female rodents. This will help provide insight into potential neural mechanisms underlying sex-specific critical windows for stress susceptibility and behavioral emergence.

## Introduction

The corticolimbic system plays a critical role in the regulation of affective behaviors and is highly conserved across mammalian species (LeDoux, 2012). In early life, the maturation of corticolimbic regions, such as the hippocampus, amygdala, and prefrontal cortex, coincides with changes in various cognitive domains, including memory, decision-making, and threat and reward processing (Kim and Richardson, 2010; Adriani et al., 2012; Callaghan et al., 2014; King et al., 2014; Alberini and Travaglia, 2017; Walker D. M. et al., 2017; Ramsaran et al., 2018; Zimmermann et al., 2019). The corticolimbic system is also heavily implicated in the etiology and pathogenesis of psychopathologies, such as depression, anxiety, and fear-related disorders (Killgore et al., 2014; Swartz and Monk, 2014; Liu et al., 2017), which show high incidence rates in early life (Kessler et al., 2005) and prominent sex differences in prevalence and symptomatology (Altemus et al., 2014; Maeng and Milad, 2015; Gobinath et al., 2017; Eid et al., 2019). Additionally, exposure to early life adversity increases the risk of developing many of these disorders in adulthood (Heim and Nemeroff, 2001; Syed and Nemeroff, 2017), underlining a strong link between the corticolimbic system in early life, stress, and mental disorders. Many studies have examined developmental changes in corticolimbic regions, but these have primarily focused on male subjects only. While sex differences in the development, prevalence, and treatment outcome of many mental disorders have been widely reported (Altemus et al., 2014; Maeng and Milad, 2015; Gobinath et al., 2017; Eid et al., 2019; Kokras et al., 2019), little is known as to whether these might be attributed to sex differences in the maturational processes and/or trajectories of the corticolimbic system. Accordingly, understanding early life changes in corticolimbic circuits in both sexes will have crucial implications to elucidating how disruptions during critical windows of development may contribute to heightened vulnerability to psychopathologies.

Given the accessibility and temporal resolution of genetic, circuit, and behavioral manipulations, rodents are a powerful tool in this avenue of research. Like in humans, both regional and circuit dynamics in the rodent corticolimbic system are susceptible to alteration by extrinsic stress (Bangasser and Valentino, 2014; Killgore et al., 2014; Fragale et al., 2016; Liu et al., 2017; Hill et al., 2018; Johnson et al., 2018; Honeycutt et al., 2020). Considering the strong relationship between stress and psychopathology (Kessler, 1997; Nestler et al., 2002; Bale, 2006; Risbrough and Stein, 2006), many studies use rat and mouse models to examine sex differences in stress-induced perturbations of neural processes and their related behavioral outcomes. While this is beyond the scope of our review, numerous comprehensive reviews illustrate not only how male and female rodents display differential stress responses in adulthood (Luine et al., 2017; Walker C. D. et al., 2017; Bangasser and Wiersielis, 2018; Wellman et al., 2018), but also how they differ in their distal and proximal stress responses depending on when stress exposure occurs in development (Hodes and Epperson, 2019). In short, stress responsivity appears to be both *age*- and *sex*-dependent. It remains unclear, however, whether stress alters corticolimbic dynamics in a manner that is also both age- *and* sex-dependent, meaning whether stress experienced at a *specific age* alters corticolimbic dynamics in a *sex-specific* manner. To answer that question, it is critical to first identify basal sex differences in the maturation of corticolimbic brain regions across development. While studies examining sex differences at developmental timepoints in the hippocampus, amygdala, and medial prefrontal cortex exist, they are generally not framed as developmental studies, which restricts their access through common keyword searches.

In this review, we consolidate research on sex differences in the rodent corticolimbic system across early development. First, we provide a description of the fundamental principles of sexual differentiation in the rodent brain, which lay a foundation for the featured work and introduce key concepts discussed in later sections. We then describe regional sex differences in the development of the hippocampus, amygdala, and medial prefrontal cortex, including their implications for the emergence of sex-typical behavioral phenotypes across development. By compiling this research, we hope to provide insight into *what* and *when* sex differences emerge in the developing corticolimbic system, and to highlight how temporal dynamics of maturational trajectories may differ in male and female rodents. Importantly, we also explore how sex differences that exist during development may only be transient in nature, and do not necessarily persist into adulthood. This may help form a better understanding of why critical windows for stress sensitivity appear to be different between sexes across ages.

### Sexual Differentiation of the Rodent Brain

Sex differences in the young and adult brain are believed to be due, in part, to the actions of gonadal hormones during development (Young et al., 1964; Arnold, 2009; McCarthy and Arnold, 2011; Lenz et al., 2012; McCarthy et al., 2017). Phoenix et al. (1959) first illustrated this concept in 1959, when they injected pregnant guinea pigs with testosterone and observed the offspring’s sexual behaviors in adulthood. Perhaps unsurprisingly to a present-day reader, they found long-lasting, permanent effects of neonatal testosterone treatment, with female offspring exhibiting male-typical sexual behaviors in adulthood. In contrast, when naïve females were injected with testosterone in adulthood, no shift to male-typical behaviors was observed (Phoenix et al., 1959). These findings were later replicated in other animal models and laid the conceptual framework for what is now known as the organizational-activational hypothesis (Young et al., 1964; Arnold, 2009).

Briefly, the organizational-activational hypothesis posits that gonadal hormones have differential effects on mammalian development at different life stages. According to this paradigm, sexual differentiation of the rodent begins in the embryo. The male embryo actively synthesizes and releases testosterone, which peaks at embryonic day (E)18 and again shortly after birth before rapidly declining during the first postnatal days (Weisz and Ward, 1980; Motelica-Heino et al., 1988; Clarkson and Herbison, 2016). This released testosterone is either (1) metabolized into 17β-estradiol by the enzyme p450 aromatase, (2) metabolized into the non-aromatizable androgen dihydrotestosterone (DHT), or (3) remains unmetabolized (MacLusky and Naftolin, 1981). The testosterone-derived estradiol then acts on estrogen receptors to “masculinize” (i.e., the enhancement of physiological processes typical of males) the developing male (Whalen and Olsen, 1981; Lenz et al., 2012), with evidence of some contributions of testosterone and DHT on androgen receptors (see hippocampus and amygdala sections for specific examples). Although estrogen receptors are nuclear transcription factors and hence regulate gene expression, they also exert actions at the membrane, and can activate certain signaling transduction pathways (for a detailed review on estradiol, see: McCarthy, 2008). In contrast, in the rodent female embryo, the absence of an increase in circulating testosterone (Slob et al., 1980; Vom Saal and Bronson, 1980; Pang and Tang, 1984; Motelica-Heino et al., 1988) and the active actions of alpha-fetoprotein, a plasma glycoprotein produced during fetal life (Andrews et al., 1982) which prevents prenatal estrogens from crossing the blood-brain-barrier in rodents (McEwen et al., 1975; Bakker et al., 2006), allows for “feminization” (i.e., the enhancement of physiological processes typical of females) to occur (Lenz et al., 2012).

The early surge in testosterone in males and the actions of its major metabolite, estradiol, are believed to exert permanent *organizational* effects on tissue structure and function. This organizational period is believed to span from 1 week prior to birth to 1 week after birth (i.e., the perinatal period) (McCarthy et al., 2017). Later in life, during puberty, a second surge of gonadal hormones occurs. Here, androgens, estrogens, and progestogens are differentially secreted in males and females and exert reversible *activational* effects on the previously organized system, thereby facilitating the expression of sex-typical physiology in adulthood (Young et al., 1964; Arnold, 2009; McCarthy and Arnold, 2011; Lenz et al., 2012; McCarthy et al., 2017).

From the perspective of today’s field, the organizational-activational theory may not be sufficient to explain all the complexities of rodent sexual differentiation. Apart from the actions of gonadal hormones, genetic and environmental factors may also contribute to the emergence of sex differences (McCarthy and Arnold, 2011; McCarthy et al., 2017), and sexual differentiation of the brain may occur through region-specific mechanisms (McCarthy and Konkle, 2005). Furthermore, gonadal hormones have been reported in some cases to exert only transient effects in early life and, surprisingly, long-term alterations in adulthood, suggesting that their actions are not as dichotomous as initially described (Arnold and Breedlove, 1985). Nonetheless, the core principle that gonadal hormones can exert distinct effects at different developmental timepoints remains well-accepted. As such, their influences can coincide with critical windows of development in the rodent brain.

## The Hippocampus

### Anatomy and Hormonal Receptor Distribution

**FIGURE 1:**
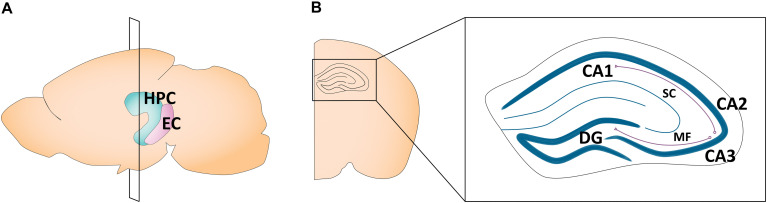
Schematic of the adult mouse hippocampus and entorhinal cortex. **(A)** Illustration in the sagittal plane depicting the adult mouse hippocampus and entorhinal cortex. HPC, hippocampus; EC, entorhinal cortex. **(B)** Illustration of the coronal view of the hippocampus at the plane of section depicted in **(A)** (bregma −1.7 mm). Hippocampal subregions most featured in the studies reviewed here are highlighted in the right panel insert. CA1, cornu ammonis 1; CA2, cornu ammonis 2; CA3, cornu ammonis 3; DG, dentate gyrus; MF, mossy fiber pathway; SC, Schaffer collateral pathway.

The hippocampus plays a critical role in learning and memory (Kandel and Spencer, 1968; Eichenbaum, 1999) and spatial navigation (O’Keefe and Dostrovsky, 1971; O’Keefe and Nadel, 1978). A number of highly comprehensive reviews have described sex differences in adult hippocampal morphology, plasticity, and its related behaviors (Jonasson, 2005; McCarthy and Konkle, 2005; Mizuno and Giese, 2010; Koss and Frick, 2017; Yagi and Galea, 2019). In humans, the hippocampus is located in the medial temporal lobe, while in rodents, it sits just beneath the neocortex (Knierim, 2015). Canonically, its structure is divided into cytoarchitectonically different subfields, notably the cornu ammonis (CA)1–4, which run along the transverse axis of the Ammon’s horn, and the dentate gyrus (Van Strien et al., 2009). The primary route of information flow is unidirectional: the dentate gyrus projects to the CA3 via the mossy fiber pathway, the CA3 projects to the CA1 via the Schaffer collateral pathway, and the CA1 projects to the entorhinal cortex, which in turn loops back to the dentate gyrus via the perforant pathway (Knierim, 2015; Figure 1). The CA1–4 and dentate gyrus can be further categorized into functionally distinct dorsal, intermediate, and ventral segments. While the dorsal hippocampus is primarily implicated in cognitive functions, the ventral hippocampus is implicated in affective processes (Fanselow and Dong, 2010; Strange et al., 2014). Apart from the CA1–4 and dentate gyrus, the greater hippocampal formation also consists of the entorhinal cortex and subiculum. In this review, our use of the term “hippocampus” refers exclusively to the CA1, CA2, CA3, and the dentate gyrus, as the featured studies did not explore other hippocampal subregions.

Sex differences in the hippocampus are at least partially modulated by the actions of gonadal hormones at androgen receptors (AR) and estrogen receptors [estrogen receptor alpha (ERα)/estrogen receptor beta (ERβ)/g-protein coupled estrogen receptor 1 (GPER1)]. In the CA1–3 and dentate gyrus, these gonadal hormone receptors are located in the dendrites, synapses, and axon terminals of principal neurons and interneurons (Loy et al., 1988; Simerly et al., 1990; Shughrue et al., 1997; Hajszan et al., 2007), as well as in glial cell processes (McEwen et al., 2012). In the adult hippocampus, some studies report equivalent expression of these receptors in males and females (Ivanova and Cordian, 2000; Hutson et al., 2019), while others find region-specific sex differences (Zhang et al., 2002). These discrepancies may be attributed to fluctuations of hippocampal AR and ERα/β expression during the estrous cycle in females, whereby sex differences emerge at certain phases, but disappear in others (Feng et al., 2010; Mitterling et al., 2010).

In the developing hippocampus, ERα levels rise and fall during the perinatal period, but inconsistencies have been noted in the precise time course. While early studies in rats found estrogen receptor expression to peak at postnatal day (P)7 in both sexes (O’Keefe and Handa, 1990; O’Keefe et al., 1995), studies that have selectively examined ERα report peak mRNA expression at P1 in mice (Mogi et al., 2015) and protein expression at P10 in rats (Solum and Handa, 2001). In the mouse hippocampus, one study reports ERβ mRNA levels to decrease from E17 to P7 in both sexes (Mogi et al., 2015), while others report them to remain relatively stable (Ivanova and Cordian, 2000; Tsai et al., 2015; Franklin et al., 2019). These inconsistencies may be due, in part, to variances in what subregions of the hippocampus were examined. Indeed, one study showed that the CA1, CA3, and dentate gyrus subregions of the mouse hippocampus each exhibits differential fluctuations in ERβ protein levels from P0 to P21 (Zuloaga et al., 2014). Meanwhile, AR mRNA levels in both the male and female mouse hippocampus increase from as early as E11 (Young and Chang, 1998), and up until P21 (Mogi et al., 2015; Tsai et al., 2015; Franklin et al., 2019).

Overall, it remains unclear whether hippocampal estrogen and androgen receptors differ by sex in early life. Some studies have reported perinatal differences in gonadal hormone receptor expression between male and female mice, but findings are variable (Ivanova and Cordian, 2000; Mogi et al., 2015), and are not observed in rats (O’Keefe and Handa, 1990; O’Keefe et al., 1995; Solum and Handa, 2001). More evidence is needed to determine whether this might be explained by species-specific sex differences. Meanwhile, circulating gonadal hormone concentrations do change in a sex-specific manner during the perinatal period, with males exhibiting a peak in testosterone at E18 and shortly after birth (Weisz and Ward, 1980; Motelica-Heino et al., 1988; Clarkson and Herbison, 2016), whereas testosterone levels remain relatively low in females (Slob et al., 1980; Vom Saal and Bronson, 1980; Pang and Tang, 1984; Motelica-Heino et al., 1988). Sex differences in circulating perinatal estradiol and androgen concentrations are thus believed to drive, at least partially, sexual differentiation in the rodent hippocampus.

### Volume and Morphology

In adulthood, male rodents generally have larger regional hippocampal volumes than females (Roof and Havens, 1992; Roof, 1993a; Madeira and Lieberman, 1995; Isgor and Sengelaub, 1998, 2003; Nuñez et al., 2000), particularly in the CA1, which is 10–16% larger in males than in females (Isgor and Sengelaub, 1998; Nuñez et al., 2003a). In mice, this volumetric sex difference emerges between P10 and P17 and persists until P65 (Qiu et al., 2018), though contradictory findings have been reported. In one study, no sex differences in CA1 and CA3 volumes were found at E17, or at P4, P30, and P60 in rats. The only observed sex difference was at P0, wherein female CA3 volume was surprisingly greater than that of males (Schwarz et al., 2012). Subtle morphological differences in the male and female rodent hippocampus, however, do seem to exist and have been more consistently reported. In adult rats, males show larger CA1 and CA3 pyramidal field volume and soma size than females (Isgor and Sengelaub, 1998), which is attributed to greater dendritic length and branch number (Isgor and Sengelaub, 2003). During development, males exhibit greater CA1 and CA3 neuron number at P7 (Hilton et al., 2003) and at P21 (Nuñez et al., 2003b), as well as glial cell count at P4 (Zhang et al., 2008), all of which may contribute to a larger volume size (Qiu et al., 2018). In contrast, the dentate gyrus does not differ in volume, density, or cell number between sexes in either young or adult rodents (Isgor and Sengelaub, 1998; Hilton et al., 2003; Schwarz et al., 2012), though the right granule cell layer is reported to be larger in width in adult males than females (Roof and Havens, 1992). Interestingly, one study found that P21 female rats have a greater number of dendritic segments on dentate gyrus granule cells than male counterparts (Juraska, 1990), and another study reported greater synaptic spine and bouton density in the CA1 of female mice compared to males at P15 (Weinhard et al., 2018). These sex differences disappeared by P60 (Juraska, 1990) and P40 (Weinhard et al., 2018), respectively. This suggests the presence of both short- and long-lasting transient sex differences in hippocampal morphology during early development, which do not necessarily persist beyond adulthood.

Notably, these morphological differences in the male and female hippocampus are influenced by the actions of estradiol and androgens in the perinatal period. In agreement with the organizational-activational hypothesis, the effects of estradiol during the perinatal period are believed to exert permanent organizational effects on tissue development and contribute to the “masculinization” of the male rodent (Young et al., 1964; Arnold, 2009; Lenz et al., 2012). In fact, early seminal studies examined the organizational hypothesis by testing the effects of manipulating estradiol levels (e.g., castration in males or estradiol/testosterone treatment in females) in the perinatal period on adult hippocampal morphology. Prenatal testosterone treatment in females is sufficient to drive greater dendritic length and branch number in the CA1 and CA3 in adulthood (Isgor and Sengelaub, 2003), which contributes to a male-typical CA1 and CA3 pyramidal field volume and soma size (Isgor and Sengelaub, 1998). Neonatal castration in males renders the opposite effect, leading to a female-typical smaller CA1 and CA3 pyramidal field volume, soma size, and dendritic length and branch number (Isgor and Sengelaub, 1998, 2003). Interestingly, while sexual differentiation has primarily been attributed to the aromatization of testosterone to estradiol in early life (Whalen and Olsen, 1981), one study reports that androgens themselves may contribute to sex differences in adult hippocampal morphology. Isgor and Sengelaub (1998) found that development of male-like CA3 morphology occurred when females were treated prenatally with testosterone or DHT, but not when treated with estradiol alone (Isgor and Sengelaub, 1998). Sexual differentiation of the rodent hippocampus may therefore begin in early life with the organizational effects of perinatal estradiol and androgens, a recurring theme in the studies discussed in this section.

Importantly, the strong organizational effects of perinatal estradiol and androgens on hippocampal morphology and behavior described above do not exclude a contribution of gonadal hormone release in later life on sex differences in adulthood. While an in-depth discussion on the effects of gonadal hormones on adult brain physiology and behavior is beyond the scope of this review (see: Spencer et al., 2008; McEwen and Milner, 2017), it is important to highlight that the release of estrogens and androgens in adulthood may induce reversible activational effects on pre-organized hippocampal networks (Mendez et al., 2011; Spritzer et al., 2011). For instance, it is well established that estrogen and androgens increase spine density in the adult hippocampus of female and male rodents, respectively (Woolley et al., 1990; Woolley and McEwen, 1992; Leranth et al., 2003). Furthermore, adult hormone treatment of testosterone in males castrated around birth, and of estradiol in females “masculinized” with estradiol in early life, is sufficient in reinstating sex-typical hippocampus-dependent behavior (Dawson et al., 1975). The effects, however, are less pronounced than those of perinatal hormonal manipulation.

The production and release of these estrogens and androgens are heavily regulated by the actions of the luteinizing hormone (LH) (Levine, 1997; Milad et al., 2009b; Christian and Moenter, 2010). Interestingly, early research on the effects of perinatal testosterone on the female reproductive system illustrates that the development of adult sex-typical patterns of LH secretion, and therefore adult sex-specific regulation of neuroendocrine function, not only depends on gonadal hormones during the early perinatal period, but during puberty as well. When treated with low doses of testosterone during the first postnatal week, female rodents exhibit normative estrous cycle after puberty, but cycles stop prematurely during adulthood in rats (Swanson and Van der Werff ten bosch, 1964; Gorski, 1968) and mice (Mobbs et al., 1985). When treated neonatally with higher doses of testosterone, animals achieve puberty but become permanently anovulatory (Swanson and Van der Werff ten bosch, 1964; Gorski, 1968). This phenomenon is referred to as the delayed anovulatory syndrome (Gorski, 1968), wherein female rats “masculinized” by neonatal testosterone lose the capacity to secrete the necessary amount of LH to trigger ovulation and consequently lose estrous cyclicity (Harlan and Gorski, 1977; Mobbs et al., 1985). Notably, if these animals are ovariectomized prior to puberty, they continue to cycle past the age at which anovulation and persistent estrus takes place (Arai, 1971). This suggests that the development of delayed anovulatory syndrome is not solely dependent on perinatal testosterone. Rather, it requires both the perinatal release of testosterone as well as the subsequent pubertal actions of estrogens and/or androgens on neuroendocrine function. This means that not only might pubertal hormones exert reversible activational effects on pre-organized systems, they might also cement sex differences induced by perinatal hormones. Therefore, while gonadal hormones in the perinatal period may heavily influence hippocampal development into sex-typical phenotypes, sex differences are still modulated by further actions of these hormones in both puberty and adulthood.

Recent findings, however, have greatly challenged our understanding of how perinatal estradiol and androgens contribute to the organizational effects of sexual differentiation and/or the emergence of transient sex differences in early life. While plasma concentrations of gonadal hormones are believed to be an accurate reflection of brain tissue levels (Pardridge and Mietus, 1979; Cornford et al., 1982; Banks, 2012), they may not capture brain region-specific differences in content, or hormones that arise from local *de novo* synthesis. Indeed, no sex differences are found in hippocampal tissue concentration levels of testosterone, estradiol, or DHT both prenatally (E19 and E21) and postnatally from P0 to P60 (Konkle and McCarthy, 2011). Furthermore, no reductions in these gonadal hormone concentrations are observed at P3 following neonatal gonadectomy, suggesting that early gonadal steroidogenesis is replaced by local steroidogenesis in the brain (Konkle and McCarthy, 2011). These findings greatly challenge most of the field’s understanding of hippocampal sexual differentiation and highlight the considerable complexities of gonadal hormone modulation in early life.

### Cell Proliferation and Cell Death

One of the most consistent sex differences in the developing hippocampus comes from studies looking at cell proliferation. As early as P1 and P4, male rats show increased cell proliferation in the CA1, CA3, and dentate gyrus compared to females (Zhang et al., 2008; Bowers et al., 2010), leading to a greater neuron number in these subregions at P7 (Hilton et al., 2003), and in CA1 and CA2/3 at P21 (Nuñez et al., 2003b). This also seems to extend to sex differences in the localization of neonatally born dentate granule cells in adulthood (Muramatsu et al., 2007), although whether this reflects changes in migration, proliferation, and/or survival remains unknown. When treated with estradiol, testosterone, or DHT shortly after birth, females exhibit an increase in the number of proliferating cells in these hippocampal subregions to a number comparable to that of males (Zhang et al., 2008; Bowers et al., 2010), which persists until at least P21 (Bowers et al., 2010), suggesting that perinatal gonadal hormones drive the increased hippocampal proliferation in males. Interestingly, neonatal treatment of males with either estradiol or testosterone has no effect on cell proliferation (Zhang et al., 2008; Bowers et al., 2010), suggesting that enhanced male-typical cell proliferation cannot be further exacerbated by increased concentrations of these gonadal hormones during the perinatal period. Furthermore, treatment with the androgen receptor antagonist, flutamide, does not alter cell proliferation in males (Zhang et al., 2008), indicating that testosterone’s actions at androgen receptors are not modulating cell proliferation in males. Although these data point to perinatal estradiol as the driver of increased hippocampal cell proliferation in males, definitive evidence (e.g., examination of the effects of male neonatal castration on perinatal cell proliferation) is still missing. Taken together, these findings highlight greater hippocampal cell proliferation in males in early life compared to females, which might be driven by the perinatal release of estradiol and androgens.

Interestingly, this sex difference in hippocampal cell proliferation does not persist into adulthood (though evidence shows sex, region, and age-dependent effects of gonadal hormones on hippocampus neurogenesis; please see Galea, 2008; Galea et al., 2013; Mahmoud et al., 2016; Yagi and Galea, 2019; Spritzer and Roy, 2020 for a more comprehensive discussion). As it pertains to the early life differences highlighted above, cell proliferation in the dentate gyrus in adulthood switches toward increased proliferation in females, but only during the proestrus phase, when estrogen levels are elevated (Tanapat et al., 1999). Furthermore, while in adult females estradiol enhances cell proliferation and decreases cell survival, it has minimal effects in males (Barker and Galea, 2008), wherein cell survival is increased by androgens (Spritzer and Galea, 2007; Swift-Gallant et al., 2018). Overall, this suggests transient sex differences in hippocampal cell proliferation in early life, and possible age-specific effects of gonadal hormones on hippocampal cell proliferation in males. In contrast to cell proliferation, cell death in the CA1, CA3, and dentate gyrus does not differ between male and female rats at P4, and it is not affected by hormonal treatments (Zhang et al., 2008), suggesting that cell proliferation, and not cell survival, is primarily driving sex differences in hippocampal neuron count in early life (Hilton et al., 2003; Nuñez et al., 2003b), and possibly contributing to volumetric disparities (Qiu et al., 2018).

### Microglia

Over the past decade, there has been a growing interest in the role of microglia in brain development and circuit maturation. Not only are microglia involved in the formation, elimination, and maturation of synapses (Paolicelli et al., 2011; Schafer et al., 2012; Parkhurst et al., 2013), but impairment in microglial function has also been implicated in neurodevelopmental disorders such as autism spectrum disorders (Zhan et al., 2014) and schizophrenia (Sekar et al., 2016). Furthermore, microglia are involved in sexual differentiation of the developing brain (for review, see VanRyzin et al., 2018; Bordt et al., 2020), though studies examining microglial sex differences in the hippocampus are few. One metric that can be affected by changes in microglia function is cell proliferation and number. Microglial phagocytosis of progenitor cells can reduce overall cell proliferation (Cunningham et al., 2013), including in the adult hippocampus (Sierra et al., 2010; Diaz-Aparicio et al., 2020). Accordingly, differences in microglial phagocytosis may contribute to sex differences in neonatal hippocampal cell proliferation. Indeed, even though both sexes display similar total microglia count in the hippocampus at P2–3, female rats display a greater proportion of phagocytic microglia compared to males (Nelson et al., 2017). This increased number of phagocytic microglia in P2–3 females can be decreased to male levels through neonatal estradiol treatment, but not DHT (Nelson et al., 2017). Interestingly, this difference in the number of phagocytic microglia is not present at E17 and E20 (Schwarz et al., 2012; Nelson et al., 2017), when there is a peak of testosterone release in the male embryo (Weisz and Ward, 1980; Schwarz et al., 2012), suggesting that estradiol may only affect microglia dynamics after birth.

Furthermore, in the mouse CA1, microglial volume and phagocytic capacity peaks at P8 in females, and at P15 in males (Weinhard et al., 2018). These measures then decrease with age in both sexes, with females exhibiting lower microglial volume and phagocytic capacity at P28, and absence of sex differences by P40 (Weinhard et al., 2018). This demonstrates an earlier rise and fall in hippocampal microglial volume and phagocytic capacity in females compared to males in early life. Integrating these data, one hypothesis is that the lower level of perinatal estradiol in females (Slob et al., 1980; Vom Saal and Bronson, 1980; Pang and Tang, 1984; Motelica-Heino et al., 1988) increases microglial phagocytosis of progenitor cells during the first postnatal week (Nelson et al., 2017; Weinhard et al., 2018), contributing to their reduced cell proliferation levels compared to males (Zhang et al., 2008; Bowers et al., 2010). Conversely, one might predict that the higher level of perinatal estradiol in males would lead to decreased microglial phagocytosis and higher baseline proliferation. Nevertheless, direct manipulation of microglial phagocytosis at these early ages is necessary to confirm whether this is the mechanism driving sex differences in hippocampal neurogenesis in early life.

### GABAergic Signaling

During early development, a switch from GABA_A_-mediated excitation to inhibition occurs in a wide array of brain structures, including the hippocampus (for a detailed review, see: Ben-Ari et al., 2007, 2012). During the prenatal and perinatal periods, high expression of the NKCC1 transporter in hippocampal pyramidal neurons maintains a high intracellular chloride concentration (Rivera et al., 1999; Kuner and Augustine, 2000; Marandi et al., 2002), leading to GABA_A_ receptor-induced membrane depolarization (Ben-Ari et al., 1989; Cherubini et al., 1991; Leinekugel et al., 1995; Ben-Ari et al., 2012; Bano and Ankarcrona, 2018). This GABA-mediated excitation precedes glutamatergic synaptic transmission and is the primary driver of hippocampal excitation in early life (Ben-Ari et al., 1989). As the brain matures, upregulation of the KCC2 transporter lowers the intracellular chloride concentration, driving GABA_A_-mediated hyperpolarization (Rivera et al., 1999; Khirug et al., 2005; Ben-Ari et al., 2007, 2012; Tyzio et al., 2008). This shift in GABAergic transmission plays an important role in the development of hippocampal circuit formation (Leinekugel et al., 1995; Ben-Ari et al., 1997; Cancedda et al., 2007; Wang and Kriegstein, 2008; Kirmse et al., 2015; Oh et al., 2016; Valeeva et al., 2016; Brady et al., 2018; Salmon et al., 2020), and its disruption is implicated in neurodevelopmental disorders (Deidda et al., 2014).

Traditionally, the developmental switch from GABA_A_-mediated excitation to inhibition in the hippocampus has been exclusively examined in male rats. In more recent years, studies incorporating females have highlighted a precocious development of GABA_A_ transmission in female rats compared to males (Table 1). In female rats, the switch from GABA_A_-mediated excitation to inhibition in CA1 and CA3 pyramidal neurons occurs sometime between P4 and P7, earlier than in males (Nuñez and McCarthy, 2003, 2007, 2008; Nuñez et al., 2003b; Perrot-Sinal et al., 2003; Galanopoulou, 2008; Damborsky and Winzer-Serhan, 2012; Murguía-Castillo et al., 2013). In male rats, the transition occurs sometime between P7 and P14 (Leinekugel et al., 1995; Rivera et al., 1999; Dzhala and Staley, 2003; Nuñez and McCarthy, 2003, 2007, 2008; Nuñez et al., 2003b; Perrot-Sinal et al., 2003; Khazipov et al., 2004; Tyzio et al., 2007; Galanopoulou, 2008; Damborsky and Winzer-Serhan, 2012; Murguía-Castillo et al., 2013), suggesting a longer window of GABA_A_-mediated excitation compared to females. Although some studies looking exclusively at male rats have reported an earlier timeline for this switch (P5 and P8, similar to females: Ben-Ari et al., 1989; Swann et al., 1989; Garaschuk et al., 1998), studies that have incorporated both sexes have consistently found females to exhibit an earlier switch, regardless of the precise age window (Table 1). One possible explanation for the variation in these timelines is that these studies used indirect metrics of assessing GABA polarity, such as NKCC1/KCC2 transporter mRNA/protein expression and muscimol-induced cell death. In the one study that performed patch-clamp recordings, the switch happened by P4 in female rat CA1 pyramidal neurons and P14 in males (Galanopoulou, 2008). In addition, these studies compare metrics between males and females at young ages, without examining adult levels. In mice, the switch occurs between P5 and P14 in males (Ludwig et al., 2003; Khirug et al., 2005), but its onset in females has not been characterized.

**TABLE 1:** Age at which GABA_A_–mediated excitation transitions into inhibition in both sexes in rats by study.

Study	Rat strain	Brain region	Method of detecting GABA polarity	Onset of GABA-mediated inhibition	
				
				F	M
Damborsky and Winzer-Serhan (2012)	Sprague Dawley	CA1 + CA3	*In situ* hybridization NKCC1 and KCC2 expression	P5	P8
Nuñez and McCarthy (2007)	Sprague Dawley	CA1 + CA3	Western Blot NKCC1 and KCC expression	P7	P14
			Muscimol-induced pCREB		
			Muscimol-induced [Ca^2+^]_*i*_		
Nuñez and McCarthy (2003)	Sprague Dawley	CA1 + CA3	Muscimol-induced cell death	P7	>P7
Nuñez et al. (2003a)	Sprague Dawley	CA1 + CA3	Muscimol-induced cell death	P7	>P7
Perrot-Sinal et al. (2003)	Sprague Dawley	CA1	Muscimol-induced pCREB	<P12	< P12
Galanopoulou (2008)	Sprague Dawley	CA1	Patch-clamp recording	P4	P14
			Immunohistochemistry KCC2 expression	P10	>P10
Lee et al. (2014)	Sprague Dawley	CA1 + CA3	Isoflurane-induced cell death	<P7	>P7
			Isoflurane-induced behavioral deficits		
Sasaki Russell et al. (2019)	Sprague Dawley	CA1 + CA3	Isoflurane-induced cell death	P4 > P7	N/A
			Isoflurane-induced behavioral deficits		
			Immunohistochemistry NKCC1 and KCC2 expression		Inconclusive

**Studies examining this transition in females (F) and males (M), broken down by rat strain, brain region and measurement of GABA polarity.**

The earlier switch in GABA polarity in females coincides with sex- *and* age-dependent increases in KCC2 levels (Nuñez and McCarthy, 2007; Galanopoulou, 2008; Damborsky and Winzer-Serhan, 2012). Interestingly, a study by Murguía-Castillo et al. (2013) found that the P1-P15 increase in KCC2 protein expression is far greater in females compared to males. In contrast, males exhibited a much larger age-dependent change in NKCC1 compared to females (Murguía-Castillo et al., 2013). Collectively, these findings suggest that the switch in GABA_A_ transmission in males might be predominantly driven by a decrease in NKCC1 expression, whereas the switch in females happens mostly as a result of an increase in KCC2 expression. The precise temporal relation between changes in NKCC1/KCC2 protein expression and respective changes in GABA_A_ signaling is yet to be established. In addition to sex differences in the timing and mechanism of the switch in GABA_A_ transmission, male and female rodents also differ in the size of the GABAergic response at birth. GABA_A_-mediated excitatory responses are greater in newborn males compared to females, and this is exacerbated by pre-treatment with either estradiol or DHT (Nuñez et al., 2005; Nuñez and McCarthy, 2008). Furthermore, prolonged estradiol treatment in males can maintain hippocampal pyramidal cells in a state of GABA_A_-mediated excitation past the normative developmental age of transition into inhibition (Nuñez et al., 2005). These data indicate that estradiol in males might potentiate the GABA_A_-mediated excitatory response and delay its switch to inhibition.

Sex differences in the maturation of GABAergic transmission in the hippocampus bear considerable implications to outcomes related to clinical exposure to drugs affecting GABAergic signaling in early life. One example is isoflurane, a commonly used anesthetic agent which binds to GABA_A_ receptors (Wang and Slikker, 2008). In young animals, isoflurane treatment leads to acute calcium overload and apoptosis in hippocampal pyramidal neurons through GABA_A_ receptor activation (Zhao et al., 2011). In male rats, exposure to isoflurane at P7 leads to acute cell death in the CA1, CA3, and dentate gyrus, as well as poorer performance in object place, object context, and social-recognition tasks in adolescence compared to female rats (Lee et al., 2014). In contrast, isoflurane treatment in P4 females leads to worse behavioral outcomes and increased cell death compared to treatment at P7 (Sasaki Russell et al., 2019), suggesting the earlier GABA switch in females may confer a protective effect against isoflurane treatment at P7 compared to males. These findings illustrate how the precocious development of GABA_A_-mediated inhibition in females may influence long-term behavioral outcomes, and highlight the importance of understanding *sex-specific critical periods* for disruption of GABAergic transmission.

### Summary and Implications of Sex Differences in the Developing Hippocampus

Studies thus far highlight both transient and long-term sex differences in the rodent hippocampus during development. While males and females differ in some hippocampal morphological features, such as dendritic length, dendritic branch number, and synaptic spine and bouton density, these differences tend to be subtle and may not persist into adulthood. Compared to females, males exhibit greater rates of cell proliferation in the CA1, CA3, and dentate gyrus in the first postnatal week, which do not persist into adulthood, wherein cell proliferation in the dentate gyrus favors females over males during the proestrus cycle. In early life, decreased cell proliferation in females may be mediated by increased microglial phagocytic capacity. Females also undergo an earlier switch from GABA_A_-mediated excitation to GABA_A_-mediated inhibition in the first postnatal week. Notably, perinatal estradiol and androgens have been consistently shown to modulate all the aforementioned sex differences, suggesting that they may play a critical role in the sexual differentiation of the rodent hippocampus. The majority of these studies have focused on male and female hippocampal development in the first two postnatal weeks. Therefore, it remains unclear (1) whether further differences emerge during puberty through the activational effects of pubertal hormones, (2) if these would be transient or long-term in nature, and (3) whether estradiol and androgens exert the same physiological and behavioral effects during puberty as they do in adulthood.

What effects might these sex differences in hippocampal maturation have on behavior? Several groups report sex differences in cognitive strategies in adult hippocampus-dependent spatial learning (Jonasson, 2005; Hawley et al., 2012; Grissom et al., 2013; Shansky, 2018; Yagi and Galea, 2019). This was assessed most prominently by examining the effects of manipulating estradiol levels in the perinatal period on adult behavior. While neonatal castration in male rats leads to female-like performance on spatial learning tasks in adulthood (Dawson et al., 1975; Joseph et al., 1978; Williams et al., 1990; Isgor and Sengelaub, 1998, 2003), this can be prevented by perinatal treatment with testosterone (Isgor and Sengelaub, 1998, 2003). In parallel, testosterone or estradiol treatment in female rats during the first postnatal week leads to male-typical behavioral patterns (Dawson et al., 1975; Stewart et al., 1975; Joseph et al., 1978; Williams et al., 1990; Roof and Havens, 1992; Roof, 1993b; Isgor and Sengelaub, 1998, 2003; Grissom et al., 2019). Importantly, these sex differences in behavior emerge as early as pre-puberty (Stewart et al., 1975; Roof, 1993a; Grissom et al., 2013, 2019), last until adulthood, and are not dependent on pubertal hormones (Joseph et al., 1978; Williams et al., 1990). While these hormones still have modulatory effects in adulthood, it is clear that their presence in early life leads to larger and more fundamental long-term effects on hippocampal function. Together with the reviewed effects of these hormones on hippocampal morphology, volume, cell number and synaptic transmission, these data further highlight the major organizational effects of estradiol and androgens on hippocampal structure and behavioral output.

A significant number of studies has shown a role for the ventral hippocampus in anxiety-like behaviors (Bannerman et al., 2004; McHugh et al., 2004; Felix-Ortiz et al., 2013; Kheirbek et al., 2013; Adhikari, 2014; Padilla-Coreano et al., 2016; Parfitt et al., 2017; Jimenez et al., 2018; Schumacher et al., 2018). Anxiety-like behavior is modulated by alterations in perinatal (Ordyan et al., 2007; Goel and Bale, 2008; Zuloaga et al., 2011; Hu et al., 2015) and pubertal gonadal hormones (Goel and Bale, 2008; Brown et al., 2015; Boivin et al., 2017; Domonkos et al., 2018; Delevich et al., 2020), and, in adulthood, by the estrous cycle (Frye et al., 2000). Despite these indications, the evidence for sex differences in anxiety-like behavior during early life is contradictory. While two studies fail to see sex differences in elevated plus maze or open field behavior at P24 and P30 (Ordyan et al., 2007; Boivin et al., 2017), one study sees transient sex differences in these behaviors at P40 (Boivin et al., 2017). Importantly, it is unclear whether and how the reported changes in hippocampal cellular maturation extend within the dorsoventral axis of the hippocampus. Future work dissecting subregion- and axis-specific hippocampal maturation in both sexes is necessary to identify possible mechanisms underlying the emergence of sex differences in hippocampal-dependent behavior. Finally, recent research tying adult sex-specific stress susceptibility to changes in the ventral hippocampus-nucleus accumbens circuit (Muir et al., 2020; Williams et al., 2020) further asserts a pressing need to expand these efforts toward the identification of sex- and circuit-specific maturation trajectories within sensitive circuits, as a means to search for potential contributions to both stress sensitivity and behavioral emergence in males and females.

## The Amygdala

### Anatomy and Hormonal Receptor Distribution

**FIGURE 2:**
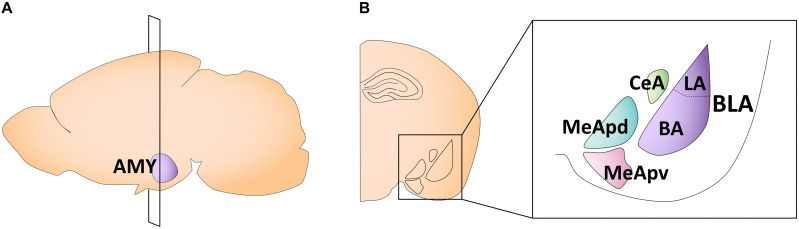
Schematic of the adult mouse amygdala. **(A)** Illustration in the sagittal plane depicting the adult mouse amygdala. AMY, amygdala. **(B)** Illustration of the coronal view of the amygdala at the plane of section depicted in **(A)** (bregma −1.7 mm). Amygdala subregions most featured in the studies reviewed here are highlighted in the right panel insert. CeA, central nucleus of the amygdala; MeApd, posterodorsal medial amygdala; MeApv, posteroventral medial amygdala; LA, lateral amygdala; BA, basal amygdala; BLA, basolateral amygdala.

The amygdala is a key structure involved in emotional learning (Davis, 1992; LeDoux, 1994). In early life, it undergoes rapid and dynamic changes in morphology, volume, cell proliferation, and physiological properties, some of which follow sex-specific patterns. The amygdala is composed of 13 subnuclei that can be broadly separated into two subdivisions: (1) the basolateral complex, consisting of the lateral, basal, and accessory basal nuclei, and (2) the centrocorticomedial group, consisting of the cortical, medial, and central nuclei (Sah et al., 2003; Hrybouski et al., 2016; Figure 2). The medial amygdala (MeA) is particularly implicated in sexually divergent behaviors, such as juvenile play, aggression, and mating (Petrulis et al., 2017). This subregion can be further divided into the anterior, posteroventral, and posterodorsal MeA (Pardo-Bellver et al., 2012; Figure 2).

Gonadal hormone receptor localization is not uniform within the amygdala. The MeA, especially within the posterodorsal subnucleus (MeApd), displays a high density of AR mRNA-labeled cells (Simerly et al., 1990) and has the highest ER count of all amygdaloid subregions (Laflamme et al., 1998). The central amygdala (CeA) also displays strong mRNA expression for gonadal hormone receptors (Simerly et al., 1990). Meanwhile, amygdala subregions that project to the cortex, such as the basolateral amygdala (BLA; encompassing the basal and lateral subnuclei) and cortical nuclei, have weaker to moderate AR and ER mRNA labeling (Pfaff and Keiner, 1973; Simerly et al., 1990; Madeira and Lieberman, 1995). Importantly, androgen and estrogen receptors can be found in both glutamatergic principal neurons and GABAergic interneurons across the amygdala, suggesting that both excitatory and inhibitory transmission can be modulated by the actions of gonadal hormones (Blurton-Jones and Tuszynski, 2002; Kiss et al., 2013). Given the prominent expression of gonadal hormone receptors in the MeA, it is unsurprising that research exploring sex differences in the amygdala has largely focused on this subnucleus. This section will therefore focus primarily on the MeA and feature studies on other amygdaloid nuclei when information is available.

### Volume, Morphology, and Synaptic Organization

In mice, females exhibit smaller overall amygdala volumes compared to males between P30 and P90, but there are no sex differences in total brain volume (Koshibu et al., 2004). When comparing different amygdala subregions in rats, the CeA and MeA reach a greater percentage of their respective adult volumes by P7 compared to the basolateral complex in both sexes (Chareyron et al., 2012). Specifically, at P7, while the lateral, basal, and accessory basal subnuclei reach 29, 44, and 33% of their adult volumes, respectively, the CeA reaches 52%, and the MeA, 77% (Chareyron et al., 2012). This suggests that the MeA matures earlier than other amygdala regions in both sexes. MeA volume increases during the first postnatal days, with female rats reaching adult-like MeA volume by P5 (Mizukami et al., 1983), whereas in males the MeA continues to gradually increase in size, reaching a volume larger than that of females by P21, which is maintained into adulthood (Mizukami et al., 1983). This is consistent with another report showing increased MeApd volumes in P26-P29 (pre-pubertal) male rats compared to females (Cooke et al., 2007). Furthermore, the larger male MeApd volume is attributed to increased neuronal number in the right hemisphere, but to more dendritic branching and overall dendritic length in the left hemisphere (Cooke et al., 2007). This volumetric sex difference may be modulated by the actions of testosterone, as male castration in adulthood leads to a decrease in MeApd volume to a size similar to females (Cooke et al., 1999). Additionally, pre-pubertal testosterone administration in male Syrian hamsters increases the volume of the posterodorsal and posteroventral MeA to adult-typical sizes (Schulz et al., 2009).

In adulthood, male rats have more MeA synapses than females (Nishizuka and Arai, 1981). This is likely driven by the organizational effects of perinatal estradiol and/or androgens, as adult sex differences disappear when females are administered with testosterone at P5 (Nishizuka and Arai, 1981). It is unclear, however, when this sex difference emerges during development. While there are no differences in MeA synapse number in male and female rats at P11 (Nishizuka and Arai, 1981), the MeApd shows signs of sex specificity and laterality in its synaptic organization in late juvenility. Between P25-P29, the left male MeApd has significantly more excitatory synapses and increased frequency of miniature excitatory postsynaptic current (mEPSC) than female rats (Cooke and Woolley, 2005). There are no sex differences in mEPSC amplitude or inhibitory synaptic transmission (Cooke and Woolley, 2005). Given that these effects are seen prior to puberty, Cooke and Woolley (2005) postulate that these sex differences in the MeApd of juvenile rats may arise from the organizational effects of perinatal gonadal hormones, consistent with what is seen in overall MeA synapse number (Nishizuka and Arai, 1981).

These organizational effects, however, do not preclude the possibility of further effects of gonadal hormone release in puberty on MeApd synaptic transmission. Indeed, pre-pubertal gonadectomy in male rats at P22, which prevents pubertal hormone release, reduces MeApd mEPSC frequency in adulthood without affecting paired pulse facilitation (and without evidence of laterality; Cooke and Woolley, 2009). Pre-pubertal castration in males also leads to decreased MeApd dendritic spine density, but with no change to overall dendritic length or branching, suggesting that pubertal gonadal hormones modulate the number of excitatory synapses in the MeApd of male rats. Overall, these findings suggest a modulatory influence of both perinatal and pubertal gonadal hormones on MeApd synaptic transmission in males. Given that no study has examined the effects of pre-pubertal gonadectomy in females on MeApd synapse number and synaptic transmission, it is unclear whether the same can be said of both sexes.

In contrast to the findings in MeA, BLA volume in rats increases between P20 and P35, and remains stable into adulthood with no evidence of sex differences in either volume or neuron number (Rubinow and Juraska, 2009). There is also no evidence of sex differences in BLA volume in young rodents (Guadagno et al., 2018; Honeycutt et al., 2020), with overall BLA volume increasing up until at least P48 in mice in both sexes (Honeycutt et al., 2020). In contrast, gonadal hormones can affect BLA spine density in a sex-specific manner. Inhibiting the conversion of testosterone to estradiol in adulthood results in decreased BLA spine density in female, but not male mice (Bender et al., 2017). Moreover, when conducted just prior to puberty, this same manipulation abolishes long-term potentiation (LTP) in juvenile female rats, while males are unaffected (Bender et al., 2017). Nevertheless, although early life stress affects BLA morphology and synaptic responses in a sex-specific way at P10-P20, there was no evidence of baseline sex differences in these metrics (Guadagno et al., 2018). Altogether, these data suggest that differences in BLA estradiol signaling can affect neuronal morphology and synaptic plasticity in females. It is unknown whether androgens modulate these parameters in males.

### GABAergic Signaling

In contrast to the hippocampus, not much is known about sex differences in GABAergic signaling in the developing amygdala. Studies indicate that the switch from GABA_A_-mediated excitatory to inhibitory transmission takes place around P21 in the BLA (Ehrlich et al., 2013), considerably later than what is seen in hippocampus (see Table 1). Importantly, even though this study incorporated both male and female rats, sex differences were not explored. In adulthood, however, there are sex differences in the number of BLA parvalbumin neurons in rats, and BLA GABAergic transmission is modulated by estrogen (Blume et al., 2017). It remains unclear, however, if these differences are already present in early life, or whether these metrics are shaped by perinatal gonadal hormones in the developing BLA.

### Microglia

In early postnatal development, particularly P0–4, male rats show more phagocytic microglia and decreased neuronal proliferation in the amygdala compared to females (VanRyzin et al., 2019). This is opposite to what occurs in the hippocampus, wherein males show less phagocytic microglia and increased cell proliferation in early life compared to females (Zhang et al., 2008; Bowers et al., 2010; Nelson et al., 2017; Weinhard et al., 2018). This perinatal difference in amygdalar microglia number and cell proliferation is affected by testosterone, as neonatal testosterone treatment on P0 and P1 in females increases phagocytic microglia and decreases cell proliferation to male-typical levels at P4 (VanRyzin et al., 2019). Notably, the greater rate of amygdalar cell proliferation seen in P4 female rats can also be reduced to male-typical levels by application of a cannabinoid receptor agonist (Krebs-Kraft et al., 2010). Given that microglia express cannabinoid receptors (Stella, 2009), the question arises as to whether gonadal hormones may interact with endocannabinoids to promote sex differences in microglia-mediated effects. Indeed, in one study, androgens were shown to enhance endocannabinoid tone and promote an increase in microglia-mediated phagocytosis in the developing amygdala (VanRyzin et al., 2019). Importantly, estradiol treatment in female pups had no effect on endocannabinoid levels, supporting the idea that gonadal hormone regulation of endocannabinoids is driven by androgens and not estradiol (VanRyzin et al., 2019). Furthermore, male rats display greater baseline endocannabinoid tone relative to females between P0 and P11 (Krebs-Kraft et al., 2010). Together, these studies suggest that modulation of endocannabinoid tone may be the link between early life androgens and sex differences in amygdalar phagocytic microglia and cell proliferation.

Interestingly, further work also links microglia activity with sex differences in juvenile play, a behavior mediated by the MeA (Meaney et al., 1981; Meaney and McEwen, 1986). In general, between weaning and puberty, male rats show greater juvenile play behavior compared to females (Argue and McCarthy, 2015). Reduction of microglia levels from P0-P4 in male rats decreases juvenile play at P27 to a level comparable to females (VanRyzin et al., 2019), indicating a direct relationship between amygdalar microglia levels and juvenile play. Moreover, neonatal activation of endocannabinoid receptors in females results in male-typical juvenile play behavior (Krebs-Kraft et al., 2010), further extending the connection between endocannabinoid tone, androgens and microglia to MeA-dependent behavior. It is important to note, however, that this early life increase in male microglia levels in the amygdala is transient. It reverses by P30, when females show more microglia than males, a difference that persists into adulthood (Schwarz et al., 2012). Unfortunately, endocannabinoid tone was not investigated at these later timepoints.

### Summary and Implications of Sex Differences in the Developing Amygdala

Most sex differences in the developing amygdala are restricted to the MeA, spanning differences in neuronal morphology, synaptic organization, cell proliferation and phagocytic microglia. In comparison to other amygdala subnuclei, the MeA shows greater expression of gonadal hormone receptors and is considered sexually dimorphic. The MeA also attains adult-typical volumes earlier than other subnuclei in both sexes, but with females reaching their adult-typical volume before males. However, by juvenility, male rats exhibit greater MeApd volumes and increased number of excitatory synapses and mEPSC frequency in the left MeApd, that are likely influenced by perinatal gonadal hormones. Pubertal gonadal hormones also influence MeA synaptic organization in males, as pre-pubertal gonadectomy leads to decreased excitatory transmission in adulthood.

Juvenile play is a prime example of the potential behavioral consequences of the sex-specific cellular and synaptic changes described in this section. Juvenile play behavior is MeA-dependent and presents prominent sex differences, with males generally displaying increased juvenile play compared to females (Olioff and Stewart, 1978; Meaney and Stewart, 1981; Auger and Olesen, 2009; Argue and McCarthy, 2015). The role of androgens in play behavior has been extensively studied, with pre-pubertal MeA lesions in male rats shown to decrease juvenile play behavior to female-like levels (Meaney et al., 1981), and, conversely, neonatal testosterone MeA administration in females increases juvenile play to a male-like frequency (Meaney and McEwen, 1986). Furthermore, perinatal application of androgen receptor antagonists in male rats decreases play behavior (Meaney et al., 1983; Hotchkiss et al., 2003), consolidating a role for neonatal androgens in the regulation of juvenile play. Further research suggests that neonatal androgens affects MeA endocannabinoid tone and phagocytic microglia, all of which directly modulate juvenile play behavior. Questions remain regarding how these microglial changes affect MeA (and related circuit) synaptic transmission to impact juvenile behavior.

In contrast to the MeA, very few studies report sex differences in the maturing BLA. The rodent BLA shows no sex differences in volume or neuron number from juvenility to adulthood, but estradiol signaling in the BLA affects synaptic plasticity in juvenile female rats. Given the sex differences in PV interneurons and inhibitory transmission in the adult rat BLA, further research is required to delineate whether BLA inhibitory transmission might be affected by perinatal gonadal hormones.

## The Medial Prefrontal Cortex

### Anatomy and Hormonal Receptor Distribution

The medial prefrontal cortex (mPFC) is heavily implicated in executive and cognitive functioning, as well as cognitive flexibility (Miller and Cohen, 2001; Vertes, 2006). Early developmental work points to adolescence as a sensitive period for the maturation of behavioral inhibition (Reynolds et al., 2019), and adolescent structural changes in the mPFC are accompanied by changes in cognitive performance and reward-related behaviors (for more comprehensive reviews, see: Brenhouse and Andersen, 2011; Walker D. M. et al., 2017). The maturation of the mPFC occurs later than other brain regions and coincides with the onset of specific behaviors relevant to emotional learning, such as developmental changes in fear learning and extinction (Miller and Cohen, 2001; Milad and Quirk, 2002; Kim and Richardson, 2007; Holmes and Wellman, 2009; Akers et al., 2012; Pattwell et al., 2012; King et al., 2014).

**FIGURE 3:**
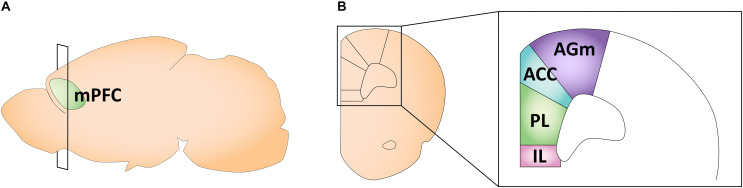
Schematic of the adult mouse medial prefrontal cortex. **(A)** Illustration in the sagittal plane depicting the adult mouse medial prefrontal cortex. mPFC, medial prefrontal cortex. **(B)** Illustration of the coronal view of the medial prefrontal cortex at the plane of section depicted in **(A)** (bregma +1.98 mm). Medial prefrontal cortex subregions most featured in the studies reviewed here are highlighted in the right panel insert. AGm, medial agranular cortex; ACC, anterior cingulate cortex; PL, prelimbic cortex; IL, infralimbic cortex.

The rodent mPFC can be divided into four distinct regions: the medial agranular (AGm), anterior cingulate (ACC), prelimbic (PL), and infralimbic (IL) cortices (Vertes, 2006; Figure 3). In the mPFC, gonadal hormone receptors are comparatively less concentrated and sparse than in other brain regions (Kritzer, 2002; Nuñez et al., 2003c). Overall, ERβ expression in the PL and IL is lower than ERα expression, and ER expression profiles do not differ by sex (Kritzer, 2002; Cover et al., 2014). Specifically, in adult rodents, the PL and IL have moderate expression of ERα and a weak expression of ERβ (Cover et al., 2014). The only study looking at AR expression in early life found relatively low AR mRNA and protein levels in the ACC (Nuñez et al., 2003c). We could not find any longitudinal studies examining gonadal hormone receptor expression across ages in the mPFC in early life. Traditionally, studies looking at different aspects of rodent mPFC maturation focused solely on males. More recent work, however, has revealed sex differences in the developmental trajectory of the mPFC, likely driven by gonadal hormones.

### Volume and Morphology

Early work investigating rat mPFC volumes across development found that in both males and females, the AGm and ACC continue to increase in size between P6 and P24, after which there is a period of decline until P30, when the volume approximates that of the adult mPFC (Van Eden and Uylings, 1985). Meanwhile, the PL reaches its maximum volume as early as P14, followed by a decrease in size at P18, with a second peak in volume at P24 and a gradual decrease through P30 into adulthood (Van Eden and Uylings, 1985). Although Van Eden and Uylings (1985) did not find sex differences in PL volume, Markham et al. (2007) found that the volume of PL/IL (combined measurement) decreases between P35 and P90 in female rats, while males did not show any change in volume between these timepoints (Markham et al., 2007). Furthermore, P90 female rats were reported to have smaller PL/IL volume than male rats (Markham et al., 2007). The same study found that in the ACC, volumes did not differ between P35 and P90 and no sex differences were found (Markham et al., 2007).

Both male and female rats exhibit a significant loss in PL/IL neurons from P35 to P90, but this decrease is more pronounced in females than males (Markham et al., 2007). A separate study from the same group found that in female rats, mPFC neuronal loss occurs between P35–45, and does not change in adulthood, while males only show a non-significant trend toward decreased neuron number from P20 to P90 (Willing and Juraska, 2015). Remarkably, pre-pubertal gonadectomy at P20 did not change the number of PL/IL neurons in males during adulthood, but did prevent the decrease in neuron number in female rats (Juraska et al., 2013; Koss et al., 2015). As such, it appears that post-pubertal changes to neuron number are more prominent in females, suggesting a stronger influence of gonadal hormones on PL/IL neuronal count.

When it comes to mPFC dendritic morphology, studies point to layer-specific sex differences in early life. Markham et al. (2013) showed that, in female rats, mPFC layer 3 dendritic complexity almost doubles between P20-P30 and further increases at P90, whereas males display a comparatively lower rate of increase across P20-P56 and no further change in adulthood. In contrast, there are no sex differences in mPFC layer 5 dendritic length or complexity, with both sexes showing an increase in these metrics between P20 and P35, which reduces by P90 (Koss et al., 2014). Interestingly, only females demonstrated a loss of layer 5 basilar dendrites between P35 and P90 (Koss et al., 2014). The authors note that similar pruning might happen in males at a slightly later timepoint that was not assessed in the study, given that male puberty is delayed compared to that of females (at P45, as opposed to around P35 in females, sampled in the study) (Korenbrot et al., 1977; Ojeda and Skinner, 2006; Castellano et al., 2011). This would imply that mPFC layer 5 basilar dendrite pruning is driven by pubertal gonadal hormone release, a conjecture that has not been directly assessed. Overall, female rats display an earlier and more dramatic period of dendritic growth in the mPFC layer 3, as well as increased pruning of layer 5 basilar dendrites between P35 and P90.

As to the contribution of gonadal hormones to mPFC structure and behavior, it is well established that estrogen administered in adulthood enhances both mPFC spine density and mPFC-dependent memory (Inagaki et al., 2012; Luine and Frankfurt, 2013). In female adult rats, ovariectomy leads to mPFC dendritic spine loss, which is reversed by estrogen administration (Luine et al., 2006; Shansky et al., 2010). Furthermore, estrogen increases dendritic spines in IL neurons that project to BLA in the adult rat (Shansky et al., 2010). Given the important role of the mPFC-BLA circuit on fear and anxiety (Likhtik et al., 2005; Sotres-Bayon and Quirk, 2010; Arruda-Carvalho and Clem, 2014; Senn et al., 2014), and the higher prevalence of anxiety disorders in females (McLean et al., 2011), these data may bear important consequences to our understanding of sex differences in the circuit basis of affective disorders. Finally, several studies also show that adult estrogen-driven changes in mPFC spines are accompanied by enhancement of behavioral performance in mPFC dependent tasks (Inagaki et al., 2012; Velázquez-Zamora et al., 2012; Luine and Frankfurt, 2013).

Despite this evidence in adulthood, it is important to emphasize that Koss et al. (2014) found no sex differences in mPFC spine density from P20-P90, suggesting that mPFC dendritic morphology, rather than spines, might be more susceptible to the effects of pubertal hormones, at least in mPFC layer 5. We were unable to find studies assessing the effects of estrogen on mPFC spines, dendritic morphology or related behavior in earlier timepoints. Finally, while both male and female rats show a decrease in mPFC synapse number following puberty, only in males is this directly linked to the emergence of physical indicators of puberty (Drzewiecki et al., 2016). This suggests that even though puberty triggers synaptic pruning in both sexes, females show a relatively slower onset in mPFC synaptic changes. Interestingly, synaptic density in the human PFC peaks around the time of puberty and decreases during adolescence for both males and females (Glantz et al., 2007). This indicates that puberty guides the onset of PFC pruning across species, in both rodents and humans.

### GABAergic Signaling

Few studies have investigated early sex differences in prefrontal GABAergic signaling. Work in male rodents point to adolescence as a significant period for the maturation of GABAergic inhibitory networks within the mPFC (see reviews: Caballero and Tseng, 2016; Zimmermann et al., 2019). Perineuronal nets, which assemble around mature interneurons that express PV (Celio and Blumcke, 1994) and are important for fear learning (Gogolla et al., 2009; Hylin et al., 2013; Xue et al., 2014; Banerjee et al., 2017), increase in male rats between P35-P36 in both the PL and IL (Baker et al., 2017). A recent study indicates that even though the number of PNNs in the PL and IL of both sexes increases between P30 and P60, puberty in females induces a transient decrease in PL PNN number around P35 (Drzewiecki et al., 2020). In mice, estrogen and progesterone influence the maturation of mPFC inhibitory transmission in female mice by shifting the balance between excitation and inhibition (Piekarski et al., 2017). Specifically, the rise in pubertal gonadal hormones coincides with an increase in inhibitory neurotransmission onto ACC pyramidal neurons (Piekarski et al., 2017). Additionally, advancing the onset of puberty accelerated this rise in inhibitory neurotransmission, such that inhibitory charge transfer and mIPSC frequency were increased to levels that normally occur in post-pubertal control females (Piekarski et al., 2017). Advancing puberty also accelerated the onset of adult-typical behavior in a working memory task (Piekarski et al., 2017). This was the first demonstration that pubertal estrogen and progesterone influence mPFC inhibitory neurotransmission and thereby affect its maturation. Collectively, these data indicate that the onset of puberty alters inhibitory processes in the mPFC of female rodents.

### Microglia

When compared to the hippocampus and amygdala, research on potential early sex differences in mPFC microglia is very limited. To our knowledge, only two studies have examined changes in mPFC microglia in development. During the perinatal period, between P0-P4, there are no sex differences in both total microglial and phagocytic microglial levels in the mPFC (VanRyzin et al., 2019). A separate study by Mallya et al. (2019) reported a rise and fall in PL microglial engulfment of dendritic spines from P24 to P50, with peak engulfment at P39 (Mallya et al., 2019). Microglial phagocytosis of presynaptic glutamatergic terminals was also more prominent at P39 than at earlier ages, and was increased still at P50 (Mallya et al., 2019). These results implicate phagocytic microglia in the pruning of PL synapses in adolescence. However, this study involved a combined sample of both male and female rats and claimed to be underpowered to detect any sex differences (Mallya et al., 2019). Future research exploring changes in mPFC microglial dynamics during peripuberty may uncover important links to the described sex-specific changes in mPFC cell number and pruning during development.

### Summary of Sex Differences in the Developing Medial Prefrontal Cortex

Converging evidence on mPFC volume, morphology, and dendritic pruning points to females demonstrating precocious maturation compared to males. These metrics change drastically following the onset of puberty in both males and females, suggesting a strong role for pubertal gonadal hormones in modulating mPFC development in both sexes. While PL/IL volume remains unchanged in male rats, females show a decrease in volume between adolescence and adulthood. During this same period, female rats demonstrate a greater loss of PL/IL neurons, suggesting a role for pubertal gonadal hormones in modulating PL/IL neuron count. Moreover, sex differences have been found in dendritic growth and pruning, with female rats exhibiting an earlier and more pronounced period of growth in mPFC layer 3 and pruning of layer 5 mPFC basilar dendrites between P35 and P90. Although little is known regarding sex differences in the maturation of mPFC inhibitory transmission, recent work suggests that the pubertal increase in estrogen and progesterone coincides with the maturation of inhibitory transmission in female mice. Likewise, the maturation of PNNs is influenced by pubertal onset in only female rats, with a notable decrease in PNNs in the PL in peripuberty. Evidence also points to adolescence as a sensitive period for the dopaminergic innervation in mPFC, impacting cognitive flexibility and behavioral inhibition (Hoops and Flores, 2017; Reynolds et al., 2018), although sex differences therein remain to be elucidated. More work is needed to uncover potential sex-specific changes in mPFC during perinatal or early juvenile periods, and whether they are affected by neonatal hormone exposure.

Medial prefrontal cortex, amygdala and hippocampus are critical components involved in emotional learning, and have been extensively described as fundamental for fear conditioning (Phillips and LeDoux, 1992; Ledoux, 2000; Maren, 2001; Sanders et al., 2003; Kim and Jung, 2006; Maren et al., 2013; Herry and Johansen, 2014; Arruda-Carvalho and Clem, 2015). While several studies fail to see sex differences in fear memory retrieval in adult rodents (Milad et al., 2009a; Rey et al., 2014; Gruene et al., 2015b; Voulo and Parsons, 2017), a growing body of evidence shows sex differences in other aspects of adult fear processing, such as generalization (Lynch et al., 2013; Keiser et al., 2017; Asok et al., 2019; Day et al., 2020), neural correlates (Baran et al., 2010; Day et al., 2020), as well as sex-specific fear behaviors such as darting (Gruene et al., 2015a; Bangasser and Wicks, 2017; Greiner et al., 2019). Indeed, interest is building on shifting efforts toward examining more subtle behavioral metrics and strategy (over ability) in rodent tasks, which may better explain sex-specific molecular and circuit signatures of seemingly equivalent behavior (Grissom and Reyes, 2019). Future work exploring the precise developmental emergence of sex-specific fear behaviors, as well as potential differences in the neural basis of fear processing during early development, are critical to contextualize the consequences of the early life changes described in this review.

A considerable literature features contradictory findings on whether adult females are more resistant to fear extinction compared to males (Baran et al., 2009, 2010; Milad et al., 2009a; Baker-Andresen et al., 2013; Fenton et al., 2014; Gruene et al., 2015a,b; Maeng and Milad, 2015; Voulo and Parsons, 2017; Velasco et al., 2019). In early life, however, two recent studies point to female-specific differences in fear processing following fear extinction. Park et al. (2017) showed that at P18, female rats already display fear renewal and spontaneous recovery following fear extinction, behaviors that were absent in P18 males. This is close to the age in which BLA displays changes in excitability (Ehrlich et al., 2012), as well as changes in mPFC-BLA transmission in male rodents (Arruda-Carvalho et al., 2017; Selleck et al., 2018), suggesting that age- and sex-specific changes within corticolimbic circuits may underlie sex differences in fear behavior in early life. Furthermore, work from the same group showed a strong correlation between high estrogen levels in P35 rats and impaired extinction (Perry et al., 2020). Interestingly, in adulthood, high estrogen levels correlate with improved fear extinction (Milad et al., 2009a; Zeidan et al., 2011), which is the opposite of what Perry et al. (2020) found in adolescence. This further underscores the importance of probing sex differences in these circuits across the lifespan. Together with the evidence on pubertal effects of estrogen and progesterone on mPFC cell and synapse number reviewed previously, adolescence emerges as a particular sensitive period to the effects of gonadal hormones on mPFC microcircuit and mPFC-dependent behavior in females.

## Concluding Remarks

The corticolimbic system is heavily involved in emotional learning (LeDoux, 2012), and impairments or disruptions to corticolimbic regions are associated with the onset and progression of affective disorders (Phillips et al., 2003; Killgore et al., 2014; Swartz and Monk, 2014; Liu et al., 2017). Provided growing epidemiological evidence of sex differences in the prevalence and symptomology of these disorders (Altemus et al., 2014; Maeng and Milad, 2015; Gobinath et al., 2017; Eid et al., 2019), it is important to first understand *what* sex differences exist in early life and *when* they emerge. Decades of prior research in neuroscience neglected the inclusion of female animals (Will et al., 2017), largely based on the assumption that circulating ovarian hormones render female subjects more variable (see Shansky, 2019 for more detail). However, physiological, cellular, and behavioral data suggest that variability within female rodents does not differ any more in magnitude than variability within males, even when factoring in the estrous cycle (Prendergast et al., 2014; Becker et al., 2016). Fortunately, sex omission in research has been declining. While the majority of our understanding of the development of corticolimbic regions pertains to the male brain only, studies that have included both male and female subjects have provided evidence of sex differences in the maturation of the hippocampus, amygdala, and medial prefrontal cortex.

In this review, we attempted to provide a longitudinal perspective of how corticolimbic regions develop in both sexes. Given that most studies span few age groups, we remain limited in our capacity to achieve a detailed longitudinal understanding of the maturational trajectories in both male and female rodents. Future work assessing behavioral and neural correlates at multiple timepoints across the lifespan in both sexes, as well as exploring the maturation of connectivity between these brain regions, is paramount to fill these gaps in knowledge. Still, it is clear that sex differences exist during early development in these three brain regions, and that they arise through a combination of different factors, including, but not limited to, activational and organizational effects of gonadal hormones, microglial processes, and early life experiences. Here, we have highlighted several lines of evidence for sex-specific regional differences in volume, morphology, synaptic organization, microglia, and GABAergic signaling, and whether they are influenced by gonadal hormones in early life.

As research continues to expand our understanding of how sex as a biological variable affects the trajectory of brain maturation and circuit formation, a key emerging question is how sex-specific maturation trajectories might shape circuit function and related behaviors. Arguably, sex specificity in the maturation of corticolimbic circuits may have implications that extend beyond early life, likely shaping sex differences in adult behavior and/or its neural correlates, as well as in the consequences of stress. Similarly, it is also conceivable that even sex differences that emerge transiently in early life, but are no longer present in adulthood, may signal sex- and brain region-specific sensitive periods underlying differential responsivity to external stimuli. Several excellent reviews have extensively described sex differences in adult rodent behavior relying on the corticolimbic circuit (Jonasson, 2005; McCarthy and Konkle, 2005; Dalla and Shors, 2009; Mizuno and Giese, 2010; Farrell et al., 2013; Koss and Frick, 2017; Velasco et al., 2019; Yagi and Galea, 2019) and in response to stress (Farrell et al., 2013; Luine et al., 2017; Walker C. D. et al., 2017; Bangasser and Wiersielis, 2018; Wellman et al., 2018; Hodes and Epperson, 2019), as well as the role of epigenetic mechanisms in the sexual differentiation of corticolimbic regions in both early life and adulthood (McCarthy et al., 2009; Matsuda et al., 2012; Zhang et al., 2013; Curley and Champagne, 2016; Mychasiuk and Metz, 2016). However, very little is known about when in development these differences emerge, and whether any of the developmental sex differences described in this review contribute to them. Furthermore, while it is tempting to speculate on how these sex-dependent developmental timelines may translate to humans, additional human and animal research is needed to draw any direct parallels. However, given the extensive human research highlighting sex differences in stress susceptibility and impacts on mental health, this rodent literature underscores the importance of studying sex as a biological variable in humans, particularly as it pertains to the maturation of corticolimbic circuits in early life.

In addition, it is important to emphasize that absence of significant or overt differences in behavioral output between males and females may be achieved through sex-specific compensatory neural mechanisms (De Vries, 2004; McCarthy and Konkle, 2005). In other words, sex differences in brain maturation do not necessarily drive sex differences in behavior. Rather, they may form part of a compensatory mechanism to *minimize* sex differences in behavioral phenotypes, despite differences in physiology and hormonal influence (De Vries and Boyle, 1998; De Vries, 2004). Altogether, it is clear that studying sex differences within (and outside of) the corticolimbic circuit generates insight into the neural basis of behavior that expands well beyond the question of how females differ from males. Widening this search to incorporate longitudinal and circuit-based perspectives will allow us to identify the intrinsic and extrinsic factors regulating the emergence of neuronal mechanisms underlying behavior, as well as sensitive periods that could inform stress sensitivity and guide sex-specific intervention strategies. In conclusion, this review reinforces the significance of expanding research on sex-specific maturational rates of the corticolimbic system and their implications in shaping behavior across the lifespan.

## Author Contributions

HP, MZ, and MA-C wrote and edited the manuscript. All authors contributed to the article and approved the submitted version.

## Conflict of Interest

The authors declare that the research was conducted in the absence of any commercial or financial relationships that could be construed as a potential conflict of interest.
